# Some Immune Parameters of Term Newborns at Birth Are Associated with the Concentration of Iron, Copper and Magnesium in Maternal Serum

**DOI:** 10.3390/nu15081908

**Published:** 2023-04-15

**Authors:** Karolina Rak, Marzena Styczyńska, Michaela Godyla-Jabłoński, Monika Bronkowska

**Affiliations:** 1 Department of Human Nutrition, Faculty of Biotechnology and Food Science, Wroclaw University of Environmental and Life Sciences, 51-630 Wrocław, Poland; 2 Institute of Health Sciences, Collegium Salutis Humanae, University of Opole, 45-060 Opole, Poland

**Keywords:** IgG antibodies, ANCA auto-antibodies against lactoferrin, mineral elements, maternal nutritional status, umbilical cord blood, pregnant women

## Abstract

The nutritional status of pregnant women is considered to affect fetal development and the health condition of newborns, including their immune system. We investigated the relationship between the concentrations of magnesium (Mg), calcium (Ca), zinc (Zn), iron (Fe) and copper (Cu) in maternal serum (MS) and the concentrations of IgG antibodies and antineutrophil cytoplasmatic auto-antibodies against lactoferrin (Lf-ANCA) in umbilical cord serum (UCS). IgG was considered as a promoter of immunity, and Lf-ANCA as an inhibitor. The examined group consisted of 98 pregnant women and their healthy term newborn children. The concentrations of mineral elements were measured by FAAS/FAES, while the concentrations of antibodies were determined by ELISA. Excessive MS Fe and insufficient MS Cu were related to insufficient UCS IgG and excessive UCS Lf-ANCA. The correlation analysis showed confirming results. Adequate UCS IgG and Lf-ANCA were related to MS Mg at the strictly lower limit of the reference values. The results obtained seem to suggest that an excess of Fe and a deficiency of Cu in pregnancy may adversely affect some immune parameters of newborns. Reference values for MS Mg are likely to require reconsideration. It would be advisable to monitor the nutritional status of pregnant women with minerals in order to support the immune capacity of newborns.

## 1. Introduction

The immune system of newborns is functionally different (not immature as was thought until recently) compared to the adult counterpart, although the acquaintance with the functional capabilities of the immune system in the first period of extrauterine life remains limited [1]. The need to maintain the mutual immune tolerance between maternal and fetal organisms during pregnancy, as well as limited prenatal contact with antigens from the external environment, promotes the immunological calming of the fetus by inhibiting the development of immune memory and the maturation of specific immunity [2]. High concentrations of immunosuppressive compounds in the blood [3], initially only naïve T and B cells (without immune memory), as well as decreased sensitivity and reactivity of immune cells to foreign antigens [4], are characteristic for newborns.

The immunity of newborns at birth is based on two pillars—specific IgG antibodies downloaded from mothers by placental transport, and elements of their own non-specific immunity [2,5].

IgG antibodies are the main class of immunoglobulins involved in the fight against pathogenic microorganisms, and they participate in the secondary immune response [6,7]. Although the immune systems of newborns produce small amounts of IgM and IgA antibodies, they are unable to produce IgG antibodies—a key element of a highly precise specific immune response [3,4]. Maternal IgG antibodies are the only ones of the five classes of immunoglobulins capable of crossing the blood–placenta barrier and entering the fetal circulatory system [7], becoming the only source of neonatal specific immunity. Additionally, they are subject to active transport, and their intensity peak is observed in the last 4 weeks of prenatal life, i.e., after the 36th week of pregnancy [5,6,8,9]. Due to the above, maternal IgG antibodies were considered in our study as a promoter of the immunity of newborns.

Elements of the non-specific immunity of newborns, despite their functional difference and decreased reactivity, nevertheless constitute the first line of defense against pathogens. A key role is played by neutrophils—the dominant population of immune cells in newborns—which, after activation, are able to release various antimicrobial proteins from their granules, including lactoferrin with an unusually broad spectrum of killing and static activity against microorganisms and various immunomodulatory properties [10,11,12,13]. Considering the crucial role of lactoferrin in the organism’s defense against infections, as well as its importance in regulating the immune response, the presence of anti-lactoferrin antibodies (Lf-ANCA) in the blood should be considered highly detrimental. These antibodies belong to the antineutrophil cytoplasmatic auto-antibodies (ANCA)—a group of auto-antibodies against antigens contained in the cytoplasm of neutrophils—and impair the proper functioning of the immune system. Although the cause of the production of Lf-ANCA in the body has not been established so far, it is known that they underline many inflammatory and autoimmune diseases [14,15,16]. Moreover, lactoferrin deficiency has been demonstrated to be associated with recurrent infections [17,18], and the administration of lactoferrin may reduce the risk of infections of various origins [19,20,21]. Due to the above, Lf-ANCA auto-antibodies were considered in our study as an inhibitor of the immunity of newborns.

In prenatal life, many factors may affect fetal development and the health condition of newborns at birth. It has been demonstrated that one of the key determinants is the nutritional status of pregnant women. An inadequacy (insufficiency, excess or imbalance of nutrients) can lead to fetal programming resulting in adverse short- and long-term health consequences in offspring, including consequences related to immunity [22,23,24,25,26,27,28,29,30,31]. Improper nutritional status in pregnant women has been recognized to be a global problem in both low- and middLe-income and high-income countries [32,33,34,35]. Its cause may be not only an insufficient supply of nutrients with increased demand [36], but also their unbalanced supply (e.g., an excess of fats and simple sugars at the expense of other nutrients) [33,37,38] and an excessive use of supplementation (especially of vitamins and minerals) [39,40].

Although there are some published results suggesting that both an excess and an insufficiency of macro- and microelements during pregnancy may lead to detrimental effects for fetal development, maternal health conditions and the health of newborn children [36,41], according to our best knowledge there are no papers that have investigated the relationship between the mineral nutritional status of pregnant women and the immune parameters of newborns at birth.

Therefore, the aim of this study was to examine the relationship between the concentration of macro- and microelements (Mg, Ca, Zn, Fe, Cu) in the blood of pregnant women and the concentration of IgG antibodies (as a promoter of immunity) and Lf-ANCA auto-antibodies (as an inhibitor of immunity) in the umbilical cord blood serum of newborns. We hypothesized that an excessive or insufficient concentration of macro- and microelements in maternal blood may negatively affect some immune parameters of their offspring at birth.

## 2. Materials and Methods

### 2.1. Patients

Ninety-eight pregnant women from the Obstetrics and Gynecology Ward of the Provincial Specialist Hospital in Wroclaw Research and Development Centre (Wroclaw, Poland) and their full-term healthy newborn children (46 boys and 52 girls) were included in the research in 2016 (April–December). Participants had uncomplicated pregnancies terminated with caesarean section (CS) due to various medical indications (i.e., tokophobia, previous CS, advanced eye defect), and all of them declared no tobacco or alcohol use throughout the pregnancy. Women with natural labors and multiple pregnancies were not included in the research. The maternal and neonatal characteristics are presented in Table 1.

### 2.2. Determination in Neonatal Cord Blood Serum of IgG Antibodies as a Promoter of Immunity

Umbilical cord blood samples were collected during CS. After clotting, blood samples were centrifuged at 2000× *g* rpm for 15 min. Serum samples (UCS) were removed and stored at −80 °C until analysis [42]. The concentration of IgG antibodies in the UCS was analyzed in duplicate using the ELISA method (Human IgG Total Ready-SET-Go! ELISA, eBioscience, Bender MedSystem GmbH, Vienna, Austria) according to the protocol recommended by the manufacturer and included in the operating instructions. An Epoch microplate spectrophotometer (BioTek Instruments, Santa Clara, CA, USA) was used to measure the absorption. Concentrations of IgG antibodies were given in mg/dL.

Based on the reference values for the concentration of IgG antibodies in the cord blood serum [43], the examined newborns were divided into two groups:

- Adequate concentration of IgG in the UCS, when it was 636–1606 mg/dL (which indicated the correct IgG-related immune functions of newborns);
- Insufficient concentration of IgG in the UCS, when it was <636 mg/dL (which indicated impaired IgG-related immune functions of newborns).

### 2.3. Determination in Neonatal Cord Blood Serum of Lf-ANCA Auto-Antibodies as an Inhibitor of Immunity

The procedure for obtaining and storing UCS samples was described in the previous paragraph. The concentration of Lf-ANCA auto-antibodies in the UCS was analyzed in duplicate using the ELISA method (Lactoferrin Ab ELISA, Demeditec Diagnostics GmbH, Kiel, Germany), according to the protocol recommended by the manufacturer and included in the operating instructions. An Epoch microplate spectrophotometer (BioTek Instruments, USA) was used to measure the absorption. Concentrations of Lf-ANCA auto-antibodies were given in U/mL.

Based on the cut-off point for the concentration of Lf-ANCA in the blood serum provided by the manufacturer in the ELISA test instructions, the examined newborns were divided into two groups:

- Adequate concentration of Lf-ANCA in the UCS, when it was <10 U/mL (i.e., the concentration of Lf-ANCA was too low to impair the immune functions of newborns);
- Excessive concentration of Lf-ANCA in the UCS, when it was ≥10 U/mL (i.e., the concentration of Lf-ANCA was high enough to impair the immune functions of newborns).

### 2.4. Determination in Maternal Serum of Selected Macro- and Microelements

Maternal blood samples were collected in the fasting state before planned CS (no labor). The procedure for obtaining and storing MS samples was the same as for the UCS. The method of determining the concentration of macro- (Ca, Mg) and microelements (Zn, Fe, Cu) in the MS has been described in detail previously [44]. The concentrations of examined macro- and microelements in the MS were categorized based on reference values for women in the 3rd trimester of pregnancy (Table 2) [45].

### 2.5. Statistical Analysis

Statistical analysis was conducted with STATISTICA (10.0). The data analyzed in our study did not meet the conditions for the use of parametric tests due to the lack of normal distributions and heterogeneity of variance in the variables tested; therefore, non-parametric tests were used. In order to estimate the association between continuous variables, Spearman’s correlation was applied. The rank between two subgroups of independent variables was compared using the Mann–Whitney test, while the variance between three subgroups of independent variables was compared using the Kruskal–Wallis test. A *p* value ≤ 0.05 was considered statistically significant for all analyses [46]. The results were presented with the use of the size of examined subgroups (N), range (min–max), correlation coefficient (rho), median value (Me), interquartile range (IQR), quartiles (Q_1–4_), value of Mann–Whitney test (MW-Z) and value of Kruskal–Wallis test (KW-H).

### 2.6. Ethical Approval

The study design and procedure were approved by the Bioethics Committee at the Medical University of Wroclaw, Poland (KB-158/2016). Written informed consent was obtained from all examined women to participate in the study together with their newborn children.

## 3. Results

The median maternal age was 33, although the age range was much wider (19–45 years) and the median value of pBMI among pregnant women was 22.1. Examined newborns were born between the 37th and 41st weeks of pregnancy, with a median gestational age of 39 weeks and a median birth weight of 3430 g (IQR 3150–3730 g). Detailed data on maternal and neonatal characteristics are presented in Table 1, together with the descriptive statistics of the concentrations of macro- and microelements in the MS and the concentrations of antibodies in the UCS of newborns.

**Figure 1:**
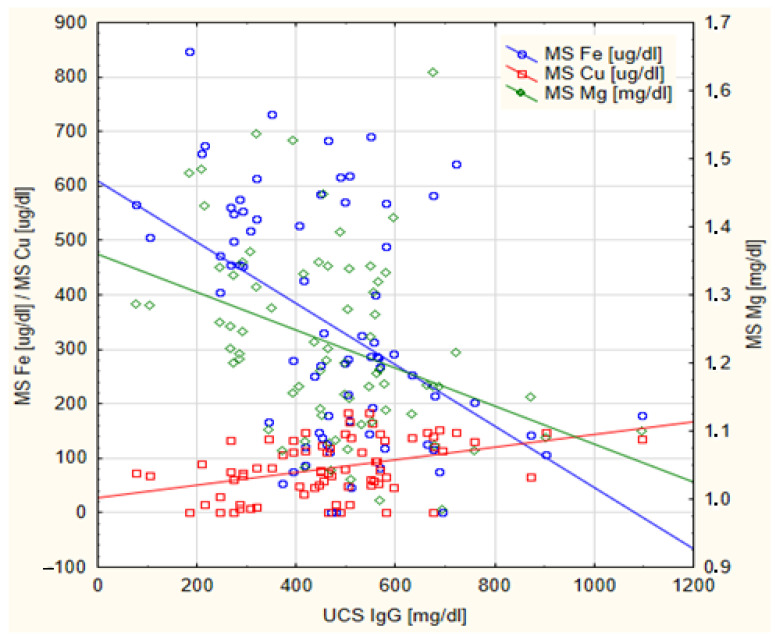
The scatterplot of the concentrations of IgG antibodies in the cord blood serum (UCS IgG) of newborns in relation to the concentration of iron (MS Fe), copper (MS Cu) and magnesium (MS Mg) in maternal serum.

**Figure 2:**
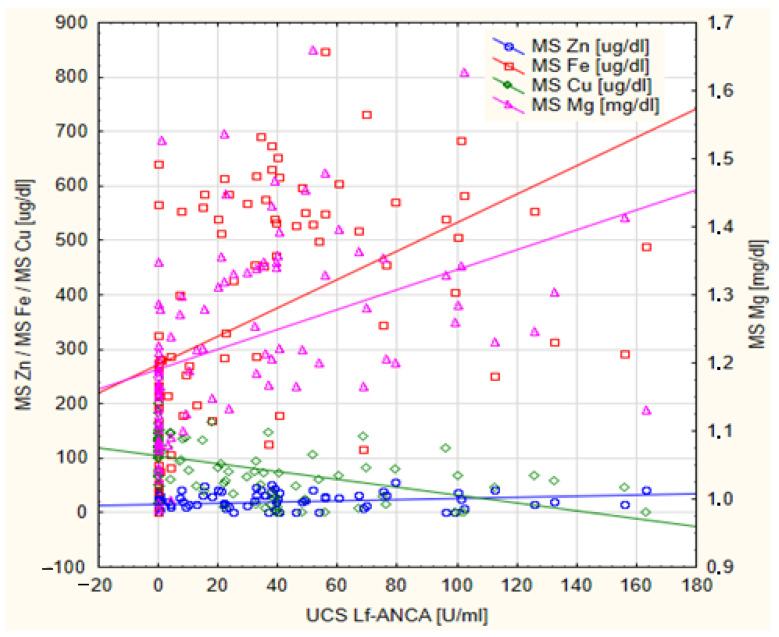
The scatterplot of the concentrations of antineutrophil cytoplasmatic auto-antibodies against lactoferrin in the umbilical cord serum (UCS Lf-ANCA) of newborns in relation to the concentration of iron (MS Fe), copper (MS Cu), zinc (MS Zn) and magnesium (MS Mg) in maternal serum.

The results of the Spearman’s correlation between the concentrations of Mg, Ca, Zn, Fe and Cu in the MS and the concentrations of IgG antibodies and Lf-ANCA auto-antibodies in the UCS are presented in Table 3. The concentration of IgG antibodies in the UCS was negatively correlated with the concentration of Mg (rho = −0.354, *p* = 0.002) and Fe (rho = −0.368, *p* < 0.001) in the MS, and positively correlated with the concentration of Cu in the MS (rho = 0.361, *p* = 0.001). Simultaneously, the concentration of these mineral elements in the MS showed the opposite direction of correlation to the concentration of Lf-ANCA auto-antibodies (rho = 0.589, *p* < 0.001; rho = 0.635, *p* < 0.001; rho = −0.613, *p* < 0.001, respectively). Additionally, a statistically significant positive correlation was observed between the concentration of Ca and Zn in the MS and the concentration of Lf-ANCA auto-antibodies in the UCS. The statistically significant correlations demonstrated in Table 3 are graphically presented in Figure 1 and Figure 2.

The median values of the concentration of Mg, Ca, Zn, Fe and Cu in the MS in relation to the concentration of IgG antibodies and Lf-ANCA auto-antibodies in the UCS (insufficient, adequate and excessive) and the results of Mann–Whitney tests between subgroups are presented in Table 4. In mothers of newborns with an insufficient concentration of IgG antibodies in the UCS, significantly higher concentrations of Mg and Fe in the MS (for Fe, excessive) and a significantly lower (insufficient) concentration of Cu in the MS were observed compared to those of newborns with an adequate level of IgG antibodies. Similarly, higher concentrations of Mg and Fe in the MS (for Fe, excessive) and a lower concentration of Cu in the MS (insufficient) were observed in mothers of newborns with an excessive concentration of Lf-ANCA auto-antibodies in the UCS compared to those of newborns with an adequate level of Lf-ANCA auto-antibodies. Additionally, in mothers of newborns with an excessive concentration of Lf-ANCA auto-antibodies in the UCS, significantly higher concentrations of Ca and Zn in the MS were demonstrated compared to mothers of newborns with an adequate level of Lf-ANCA auto-antibodies.

The concentration of IgG antibodies and Lf-ANCA auto-antibodies in the UCS in relation to the concentration of selected mineral elements in the MS (insufficient, adequate and excessive) and the results of Mann–Whitney tests between subgroups are presented in Table 5 (UCS IgG) and Table 6 (UCS Lf-ANCA). A significantly higher concentration of IgG antibodies and a lower concentration of Lf-ANCA auto-antibodies were observed in the UCS of newborns born to mothers with an adequate concentration of Fe and Cu in the MS compared to those born to mothers with an excessive concentration of Fe and an insufficient concentration of Cu in the MS. Additionally, a significantly lower concentration of Lf-ANCA auto-antibodies in the UCS (specifically, the lack of them) was demonstrated in newborns of mothers with an insufficient concentration of Mg in the MS compared to those of mothers with an adequate level of this mineral element.

Due to a great proportion of an excessive concentration of Ca (98.8%) and an insufficient concentration of Zn (98.8%) in the examined group of women, the concentration of these mineral elements was divided into quartile subgroups, as a post hoc procedure. The concentrations of IgG antibodies and Lf-ANCA auto-antibodies in the UCS in relation to the concentrations of Ca and Zn in the MS (Q_1_, Q_2_, Q_3_, Q_4_) and the results of Kruskal–Wallis tests between subgroups are presented in Table 7 (UCS IgG) and Table 8 (UCS Lf-ANCA). No significant differences in the concentration of IgG antibodies in the UCS were observed in relation to the concentration of Ca and Zn in the MS. However, the lowest concentration of Lf-ANCA auto-antibodies in the UCS, equal to 0.00 U/mL, was shown in newborns of mothers with a concentration of Ca and Zn in the MS in the range of Q_1_ compared to those of mothers with a concentration of these mineral elements in Q_2_ (for Ca) and Q_3_ and Q_4_ (for Zn).

## 4. Discussion

### 4.1. Iron

In the course of our study, a relationship of excessive MS Fe with a reduced immune capacity resulting from decreased UCS IgG and increased UCS Lf-ANCA was observed. According to the best of our knowledge, there are no similar research results in the literature for comparison.

The literature is dominated by reports on iron deficiency and iron deficiency anemia (IDA). This is a global problem [47], especially common in pregnant women [48], and it has been shown to be associated with impaired immune functions [49].

The issue of over-saturation with iron is less known as the body has a very efficient mechanism for maintaining iron homeostasis [50] and its excess occurs only as a result of disease (e.g., hereditary haemochromatosis) or excessive supplementation. Iron-containing medical products are commonly used by pregnant women suffering from iron deficiency or IDA to optimize their iron nutritional status; however, prolonged treatment may lead to its excessive concentration in the body and adverse immune effects [51]. An increased concentration of iron in the blood has been demonstrated to inhibit the Th_1_ specific response, promote an inverted ratio of CD4+/CD8+ T cells and reduce the activity of NK cells [49].

The negative impact of over-saturation with iron on immune functions can be demonstrated by an increased susceptibility to infections in patients with hereditary haemochromatosis [52]. Similarly, the administration of iron-containing supplements to infants and children despite their adequate nutritional status regarding this mineral element enhances the risk and duration of infections [53,54,55,56]. A greater susceptibility to infections accompanying an over-saturation with iron may be associated with several mechanisms—the facilitated availability of iron for the development of pathogens [57], the increased production of reactive oxygen and nitrogen species resulting in oxidative stress [58], an impaired ability of macrophages to phagocytose as a result of their over-saturation with iron [52], the suppression of enzymes involved in immune mechanisms [59] and other changes in the functioning of the immune system [49].

The results obtained in our study seem to suggest that maternal over-saturation with iron may be a potential risk factor for neonatal infections; therefore, it would be advisable to monitor the concentration of iron in the blood of pregnant women, especially in those treated with iron-containing medical products due to iron deficiency or IDA, to limit potential adverse effects on immune functions.

### 4.2. Copper

As in the case of insufficient or excessive saturation with iron, an inadequate concentration of copper in the blood is associated with impaired immune functions [49,60]. People with copper levels near the lower reference limit have been shown to have reduced lymphocyte counts [49]. Additionally, the relationship of copper deficiency to neutropenia, the impaired functioning of neutrophils, limited antimicrobial activity and an increased susceptibility to infections has been demonstrated in animal models [60].

The results of our study seem to support these findings, as significantly lower UCS IgG and higher UCS Lf-ANCA were observed in newborns of mothers with an insufficient MS Cu compared to those born to mothers with an adequate level of this mineral element. According to the best of our knowledge, there are no reports in the literature on the relationship between maternal copper nutritional status and the immune functions or the risk of infections of the offspring. So far, only the effect of an insufficient copper concentration in the blood of pregnant women has been demonstrated on other health complications in mothers and fetuses, such as pre-eclampsia [61,62], gestational hypertension [63], congenital malformations of the fetus [64], intrauterine growth restriction (IUGR) [65] or premature birth [66], the causes of which are believed to be the oxidative stress resulting from the impaired activity of Cu-dependent antioxidant enzymes [67,68,69].

### 4.3. Magnesium

In our study, MS Mg in the examined pregnant women was negatively correlated with UCS IgG and positively correlated with UCS Lf-ANCA. Moreover, significantly higher UCS IgG and significantly lower Lf-ANCA were observed in newborns of mothers with an insufficient MS Mg compared to those born to mothers with an adequate nutritional status of this mineral element. It has also been found that MS Mg exceeding 1.15 mg/dL is related to a deficiency of UCS IgG and excess UCS Lf-ANCA. Our results therefore seem to suggest a potential adverse impact of magnesium, even in the range of reference values, on the immune capacity of newborns.

According to the best of our knowledge, there are no similar research results in the literature for comparison. It is known, however, that fetal exposure to magnesium sulphate (MgSO_4_)—commonly used in pregnant women in the prevention of pre-eclampsia, preterm labor and cerebral palsy [70]—may be associated with adverse consequences in newborns, the direct cause of which may be seen in an increased concentration of magnesium in both maternal and neonatal blood [71].

A significant correlation has been demonstrated for the dose of magnesium administered to pregnant women before labor and the concentration of magnesium in their blood with the concentration of this mineral element in the blood of newborns and the health effects observed in them [72]. Additionally, an increased frequency of hypotension [73,74,75], intraventricular hemorrhage (IVH) [76] and hospitalization in the neonatal intensive care unit (NICU) [77,78,79] has been observed in the newborns of mothers treated with MgSO_4_ in pregnancy. Interestingly, in a study on preterm neonates, adverse health effects were demonstrated in both groups of newborns with the highest (≥4.5 mg/dL) and lowest (<2.5 mg/dL) levels of magnesium in the blood; however, more serious effects were observed in the former (increased incidence of both IVH and periventricular leukomalacia (PVL)) compared to the latter (increased incidence of only IVH), while the neuroprotective effect of antenatal exposure to MgSO_4_ seemed to be related to the neonatal concentration of magnesium in the range 2.5–4.5 mg/dL [72]. It seems, therefore, that an excess of magnesium compared to its insufficiency may be more harmful to newborns. This is also confirmed by the results of studies on neonates with extremely low birth weight (ELBW) [80]. The concentration of magnesium in the blood at birth of newborns who died or showed neurological development disorders at the age of 9 months was significantly higher compared to those who survived and were characterized by normal neurological development (median [IQR]: 1.7 [1.55–2.1] vs. 1.5 [1.4–1.68] mg/dL).

The results of our study seem to be consistent with the literature cited above, related to the potentially adverse effects of magnesium on the health of newborns. Moreover, they suggest that reference values for MS Mg in pregnant women in the third trimester [46] are likely to require reconsideration towards lowering them; however, extensive research is needed on this issue.

### 4.4. Zinc

In our study, a positive correlation between MS Zn and UCS Lf-ANCA was observed and an adequate UCS Lf-ANCA was related to a profound maternal deficiency of zinc, while a milder maternal deficiency of zinc was associated with excessive UCS Lf-ANCA. Our results seem to suggest a potential adverse effect of zinc on the immune capacity of newborns that is inconsistent with the literature reports on the key role of zinc in immunity.

Zinc has been shown to have immunomodulatory properties and play a key role in maintaining normal immune functions, and its deficiency impairs the mechanisms of both innate and acquired immunity, including the production of antibodies, in particular IgG [81]. Additionally, it has been demonstrated in animal models that zinc deficiency during pregnancy results in a reduced concentration of IgM, IgA and IgG antibodies in the blood serum of the offspring, and this detrimental effect may persist (in an increasingly milder form) up to the third generation [82,83]. Similarly, an impaired cellular and humoral immune response to vaccination was observed in pups born to females fed with a low-zinc diet [84].

Reports on the impact of the zinc nutritional status of pregnant women on the immunity of their offspring are limited and concern mainly prenatal supplementation with this mineral element, not the saturation of the mother’s body. Zinc supplementation in pregnancy has been demonstrated to result in an increased concentration of IgG antibodies in neonates at birth [85], an increased immune response to vaccinations in infancy [86], a reduced risk of delayed hypersensitivity to a tuberculin test (PPD) [87] and a reduced risk of diarrhea [88,89,90], dysentery and impetigo [90] in infancy. The above results suggest the positive effect of prenatal exposure to zinc on shaping immune capacity, which contradicts our results. Inconsistency, however, could be caused by the very high proportion of zinc deficiency (96.9%) in the women examined in our study. Moreover, the cited literature concerned mothers and children from developing countries (Peru and Bangladesh), where there is a common problem of malnutrition with micro- and macronutrients; therefore, zinc supplementation in these women probably only optimized their nutritional status of this mineral element. Routine dietary supplementation with zinc in pregnancy is not recommended as its excess, like its deficiency, may adversely affect the immune functions of the offspring, as has been demonstrated in animal models [91,92].

Interestingly, in the examined study group, the coexistence of common zinc deficiency in mothers (96.9%) with a common deficiency of IgG antibodies in their newborn children (83.1%) was observed. These findings seem to confirm that zinc may be essential for the placental transport of antibodies, and its perinatal deficiency may limit the acquisition of maternal antibodies by the fetus and impair the development of immunity in early life [93].

### 4.5. Calcium

In our study, a positive correlation between MS Ca and UCS Lf-ANCA was demonstrated. However, a slight deterioration of the examined immune parameters of newborns was observed after exceeding MS Ca of 16 mg/dL (>Q_1_), although a significant increase in UCS Lf-ANCA was observed only in newborns whose mothers were characterized by MS Ca in the range of Q_2_ compared to Q_1_.

According to the best of our knowledge, there are no similar research results in the literature for comparison and there is little known about the impact of the calcium nutritional status of pregnant women on the immune capabilities of their offspring. It has only been shown that prenatal exposure to maternal calcium deficiency (and anemia) does not affect the immune response in infants [94]. On the other hand, a reduced risk of death in the neonatal period has been demonstrated in newborns whose mothers were supplied daily with at least 741 mg of calcium (Q_4_) [95]. The authors suggested that this could be an effect of the reduced risk of premature birth. However, the reduced risk of death in these newborns might also be a result of a supportive effect of calcium on their immunity, as they were highly exposed to parasitic infections and malaria (Tanzania).

## 5. Conclusions

In conclusion, a significant relationship between the mineral element nutritional status in pregnant women and the immune parameters in their term newborn children has been demonstrated. An excessive concentration of Fe and an insufficient concentration of Cu in the blood serum of pregnant women in the third trimester seemed to impair the immune capacity of newborns, significantly decreasing the concentration of IgG antibodies (as a promoter of immunity) and increasing the concentration of Lf-ANCA auto-antibodies (as an inhibitor of immunity) in cord blood. Moreover, reference values for MS Mg in pregnant women in the third trimester are likely to require reconsideration towards lowering them, as MS Mg exceeding 1.15 mg/dL, although in the strictly lower limit of reference values, was related to a deficiency of UCS IgG and excess UCS Lf-ANCA. Our results concerning the relationship between the zinc and calcium nutritional status of pregnant women and the immunity of newborns are inconclusive.

The results of our study showed the need to develop strategies to optimize the mineral element nutritional status of pregnant women, as this is likely to improve the immune capacity of newborns and reduce the risk of neonatal infections and further adverse health effects in the offspring.

However, the presented results and conclusions should be confirmed in further studies. We would recommend conducting them on a larger study group of mother–newborn pairs, exceeding the minimum sample size, which would make the results scientifically credible and allow their generalization to the entire population of pregnant women and their newborn children. It could be also desirable to control the potential confounding factors that may affect the examined immune parameters of newborns, i.e., maternal age, BMI, immune health condition of mothers, maternal diet and use of an adjusted multivariate model. Future studies could also include a broader spectrum of immune parameters measured to assess the potential of neonatal immune defenses, e.g., the level and activity of NK cells, T cells or complement system. Additionally, besides the measurements of immunological markers such as the concentration of antibodies or immune-active compounds in the blood, the frequency (and severity) of neonatal infections could be registered in order to verify their correlation.

Further research is needed before these results can be implemented in nutritional recommendations for pregnant women aimed at improving the immune capacity of newborns. At this stage, our results should be considered as an important signpost for future investigations.

## Figures and Tables

**Table 1:** Maternal and neonatal characteristics (N = 98).

	N	Me	IQR	Min–Max
**Maternal characteristics**				
Mother’s age [years]	98 ^1^	33.0	30.0–35.0	19.0–45.0
Pre-pregnancy BMI [kg/m^2^]	98	22.1	20.1–25.1	18.0–32.9
MS Mg [mg/dL] ^2^	98	1.22	1.13–1.33	0.99–1.66
MS Ca [mg/dL]	98	17.68	15.93–18.96	10.18–26.94
MS Zn [µg/dL]	98	18.30	1.00–32.10	0.001–56.30
MS Fe [µg/dL]	98	331.0	167.0–555.0	0.001–1603.0
MS Cu [µg/dL]	98	75.85	38.00–122.5	0.000–248.10
**Neonatal characteristics**				
Gestational age [weeks]	98	39.0	38.0–39.0	37.0–41.0
Birth weight [g]	98	3430.0	3150.0–3730.0	2510.0–4980.00
UCS IgG [mg/dL]	77 ^2^	480.0	370.0–568.0	78.0–1477.0
UCS Lf-ANCA [U/mL]	98	17.70	0.001–47.80	0.001–163.10

MS—maternal serum, UCS—umbilical cord serum, IgG—IgG antibodies, Lf-ANCA—ANCA auto-antibodies against lactoferrin, Mg—magnesium, Ca—calcium, Zn—zinc, Fe—iron, Cu—copper, SD—standard deviation, Me—median, IQR—interquartile range. ^1^ N = 98—female newborns (♀ N = 52), male newborns (♂ N = 46). ^2^ N = 77 due to lack of data on the concentration of IgG antibodies in 21 samples of cord blood serum (11 female samples, 10 male samples); however, there were no statistically significant differences in any of the maternal or neonatal characteristics parameters between the study groups of N = 98 and N = 77 (*p* > 0.05), and sex proportions were preserved.

**Table 2:** Reference values of the concentration of examined macro- and microelements in blood serum for women in the 3rd trimester of pregnancy [45].

Examined Elements in the MS	Insufficient	Adequate	Excessive
Mg [mg/dL]	<1.1	1.1–2.2	>2.2
Ca [mg/dL]	<8.2	8.2–9.7	>9.7
Zn [µg/dL]	<50	50–77	>77
Fe [µg/dL]	<30	30–193	>193
Cu [µg/dL]	<130	130–240	>240

MS—maternal serum, Mg—magnesium, Ca—calcium, Zn—zinc, Fe—iron, Cu—copper.

**Table 3:** Spearman’s correlation between the concentration of macro- and microelements in the MS and the concentration of IgG antibodies and Lf-ANCA auto-antibodies in the UCS of newborns.

Examined Elements in the MS	UCS IgG [mg/dL] (N = 77)		UCS Lf-ANCA [U/mL] (N = 98)	
	Rho	*p*	Rho	*p*
Mg [mg/dL]	−0.354	0.002 *	0.589	<0.001 *
Ca [mg/dL]	0.060	0.606	0.214	0.034 *
Zn [ug/dL]	−0.198	0.084	0.358	<0.001 *
Fe [ug/dL]	−0.368	<0.001 *	0.635	<0.001 *
Cu [ug/dL]	0.361	0.001 *	−0.613	<0.001 *

MS—maternal blood serum, UCS—umbilical cord blood serum, IgG—IgG antibodies, Lf-ANCA—ANCA auto-antibodies against lactoferrin, Mg—magnesium, Ca—calcium, Zn—zinc, Fe—iron, Cu—copper, rho—correlation coefficient. * statistical significance *p* ≤ 0.05.

**Table 4:** Median values of the concentration of macro- and microelements in the MS in relation to the concentration of IgG antibodies and Lf-ANCA auto-antibodies in the UCS (insufficient, adequate and excessive).

Examined Elements in the MS ^3^	UCS IgG [mg/dL] ^1^		MW-Z	*p*	UCS Lf-ANCA [U/mL] ^2^		MW-Z	*p*
	Adequate (N = 13)	Insufficient (N = 64)			Adequate (N = 45)	Excessive (N = 53)		
	Me (IQR)	Me (IQR)			Me (IQR)	Me (IQR)		
Mg [mg/dL]	1.15 (1.09–1.17)	1.23 (1.15–1.33)	1.992	0.046 *	1.13 (1.08–1.19)	1.31 (1.22–1.36)	−6.195	<0.001 *
Ca [mg/dL]	16.58 (14.49–20.07)	17.57 (15.96–18.96)	0.360	0.719	16.73 (13.13–18.67)	17.88 (17.13–19.00)	−2.032	0.042 *
Zn [ug/dL]	10.50 (6.20–20.90)	17.65 (0.50–32.80)	1.550	0.121	10.20 (0.00–21.85)	24.60 (12.10–36.40)	−3.663	<0.001 *
Fe [ug/dL]	178.0 (117.0–253.0)	366.0 (174.5–564.0)	1.992	0.046 *	146.50 (84.50–270.00)	540.00 (427.0–596.0)	−6.549	<0.000 *
Cu [ug/dL]	134.90 (114.0–147.80)	71.05 (39.90–112.65)	−2.584	0.010 *	120.30 (97.80–145.10)	49.60 (11.40–73.70)	6.120	<0.001 *

MS—maternal blood serum, UCS—umbilical cord blood serum, IgG—IgG antibodies, Lf-ANCA—ANCA auto-antibodies against lactoferrin, Mg—magnesium, Ca—calcium, Zn—zinc, Fe—iron, Cu—copper, Me—median, IQR—interquantile range, MW-Z—value of Mann–Whitney test. ^1^ UCS IgG—adequate: 636–1606 mg/dL, insufficient ≤ 636 mg/dL; ^2^ Lf-ANCA—adequate: <10 U/mL, excessive: ≥10 U/mL. ^3^ Reference values: Mg 1.1–2.2 mg/dL, Ca 8.2–9.7 mg/dL, Zn 50–77 μg/dL, Fe 30–193 μg/dL, Cu 130–240 μg/dL. * statistical significance *p* ≤ 0.05.

**Table 5:** The concentration of IgG antibodies in the UCS in relation to the concentration of macro- and microelements in the MS (insufficient, adequate and excessive).

Examined Elements in the MS	UCS IgG [mg/dL]									MW-Z	*p*
	Insufficient			Adequate			Excessive				
	N	Me	IQR	N	Me	IQR	N	Me	IQR		
Mg [mg/dL]	13	507.00	463.00–686.50	64	462.50	313.00–559.50	0	---	---	−1.766	0.077
Ca [mg/dL]	0	---	---	0	---	---	77	469.00	351.00–564.00	---	---
Zn [µg/dL]	76	468.50	348.00–566.00	1	---	---	0	---	---	---	---
Fe [µg/dL]	3	---	---	22	509.00	445.00–662.00	52	451.50	289.00–557.50	2.223	0.026 *
Cu [µg/dL]	57	450.00	307.00–548.00	20	560.00	499.50–681.50	0	---	---	−3.166	0.002 *

MS—maternal blood serum, UCS—umbilical cord blood serum, IgG—IgG antibodies, Mg—magnesium, Ca—calcium, Zn—zinc, Fe—iron, Cu—copper, Me—median, IQR—interquantile range, MW-Z—value of Mann–Whitney test. * statistical significance *p* ≤ 0.05.

**Table 6:** The concentration of Lf-ANCA auto-antibodies in the UCS in relation to the concentration of macro- and microelements in the MS (insufficient, adequate and excessive).

Examined Elements in the MS	UCS Lf-ANCA [U/mL]									MW-Z	*p*
	Insufficient			Adequate			Excessive				
	N	Me	IQR	N	Me	IQR	N	Me	IQR		
Mg [mg/dL]	16	0.00	0.00–0.00	82	30.70	0.00–55.50	0	---	---	−4.136	<0.001 *
Ca [mg/dL]	0	---	---	0	---	---	98	18.75	0.00–47.80	---	---
Zn [µg/dL]	95	21.45	0.00–48.90	3	---	---	0	---	---	---	---
Fe [µg/dL]	2	---	---	29	0.00	0.00–4.30	67	36.95	12.70–64.40	−5.007	<0.001 *
Cu [µg/dL]	75	32.80	0.00–55.50	22	0.00	0.00–7.95	1	---	---	3.687	<0.001 *

MS—maternal blood serum, UCS—umbilical cord blood serum, Lf-ANCA—ANCA auto-antibodies against lactoferrin, Mg—magnesium, Ca—calcium, Zn—zinc, Fe—iron, Cu—copper, Me—median, IQR—interquantile range, MW-Z—value of Mann–Whitney test. * statistical significance *p* ≤ 0.05.

**Table 7:** The concentration of IgG antibodies in the UCS in relation to the concentration of Ca and Zn in the MS divided into quartiles.

Examined Elements in the MS	UCS IgG [mg/dL]												KW-H	*p* *
	Q_1_			Q_2_			Q_3_			Q_4_				
	N	Me	IQR	N	Me	IQR	N	Me	IQR	N	Me	IQR		
Ca [mg/dL]	20	460.0	345.0–576.0	19	467.0	272.0–548.0	19	462.0	292.0–549.0	19	487.0	413.0–597.0	3.250	0.354
Zn [µg/dL]	20	461.5	409.5–541.0	19	547.0	403.0–632.0	19	505.0	418.5–624.0	19	356.0	267.0–503.0	7.946	0.047 ^1^

MS—maternal blood serum, UCS—umbilical cord blood serum, IgG—IgG antibodies, Ca—calcium, Zn—zinc, Me—median, IQR—interquantile range, KW-H—value of Kruskal–Wallis test. * statistical significance *p* ≤ 0.05. ^1^ Tukey’s post hoc tests demonstrated no statistically significant differences in pairs of quartiles. For MS Ca (N = 77): Q_1_ ≤ 15.93; 15.93 < Q_2_ ≤ 17.55; 17.55 < Q_3_ ≤ 18.97; Q_4_ > 18.97 mg/dL. For MS Zn (N = 77): Q_1_ ≤ 1.0; 1.0 < Q_2_ ≤ 15.8; 15.8 < Q_3_ ≤ 31.1; Q_4_ > 31.1 µg/dL.

**Table 8:** The concentration of Lf-ANCA auto-antibodies in the UCS in relation to the concentration of Ca and Zn in the MS divided into quartiles.

Examined Elements in the MS	UCS Lf-ANCA [U/mL]												KW-H	*p*
	Q_1_			Q_2_			Q_3_			Q_4_				
	N	Me	IQR	N	Me	IQR	N	Me	IQR	N	Me	IQR		
Ca [mg/dL]	23	0.00 ^∆^	0.00–23.70	25	34.30 ^∆^	9.00–55.50	25	19.80	0.80–47.80	25	24.80	0.00–40.60	11.628	0.009 *
Zn [µg/dL]	25	0.00 ^†◊^	0.00–24.80	24	22.03	4.18–51.10	25	35.10 ^†^	3.25–55.50	24	36.00 ^◊^	19.8–51.70	15.034	0.002 *

MS—maternal blood serum, UCS—umbilical cord blood serum, Lf-ANCA—ANCA auto-antibodies against lactoferrin, Ca—calcium, Zn—zinc, Me—median, IQR—interquantile range, KW-H—value of Kruskal–Wallis test. * statistical significance *p* ≤ 0.05. For MS Ca (N = 98): Q_1_ ≤ 16.08; 16.08 < Q_2_ ≤ 18.89; 18.89 < Q_3_ ≤ 20.86; Q_4_ > 20.86 mg/dL; ^∆^ *p* = 0.009; For MS Zn (N = 98): Q_1_ ≤ 10.8; 10.8 < Q_2_ ≤ 28.0; 28.0 < Q_3_ ≤ 40.9; Q_4_ > 40.9 µg/dL; ^†^ *p* = 0.015; ^◊^ *p* = 0.003.

## Data Availability

Data are available on reasonable request from the corresponding author.
